# Cross-sectional survey of surrogate decision-making in Japanese medical practice

**DOI:** 10.1186/s12910-021-00698-0

**Published:** 2021-09-24

**Authors:** Masashi Tanaka, Seiji Bito, Aya Enzo, Takethoshi Okita, Asai Atsushi

**Affiliations:** 1 Department of Clinical Epidemiology, National Hospital Organization Tokyo Medical Center, 2-5-1 Higashigaoka, Meguro-ku, Tokyo, 152-8902 Japan; 2 Department of Medical Ethics, Tohoku University Graduate School of Medicine, 2-1 Seiryotyou, Aoba-ku, Sendai, Miyagi 980-8575 Japan; 3 KARADA Internal Medicine Clinic Shibuya, 1-23-13 Jinnan, Shibuya-ku, Tokyo, 150-0041 Japan

**Keywords:** Older adults, Japan, Cross-sectional survey, Surrogate decision-making

## Abstract

**Background:**

Instances of surrogate decision-making are expected to increase with the rise in hospitalised older adults in Japan. Few large-scale studies have comprehensively examined the entire surrogate decision-making process. This study aimed to gather information to assess the current state of surrogate decision-making in Japan.

**Methods:**

A cross-sectional survey was conducted using online questionnaires. A total of 1000 surrogate decision-makers responded to the questionnaire. We examined the characteristics of surrogate decision-makers and patients, content of surrogate decision-making meeting regarding life-sustaining treatment between the doctors and surrogate decision-makers, extent of involvement of the various parties in the surrogate decision-making process, judgement grounds for surrogate decision-making, and frequency of involvement in the surrogate decision-making process.

**Results:**

Of the participants, 70.5% were male and 48.3% were eldest sons. Only 7.6% of the patients had left a written record of their preferences and 48.8% of the surrogates reported no knowledge of the patient having expressed their prior intentions regarding medical care in any form. Respondents indicated that their family meetings with healthcare professionals mostly included the information recommended by guidelines in a surrogate decision-making meeting in Japan. Most participants reported a good understanding of the meeting content. Although many participants based their decisions on multiple grounds, surrogates’ considerations may not adequately reflect respect for patient autonomy in Japan. Specifically, the eldest son considered his own preference more frequently than that of the other surrogate decision-makers. In 26.1% of the cases, either zero or one family meeting with healthcare professionals was held. In these cases, significantly fewer decisions involved the participation of healthcare professionals other than the doctor compared to cases with multiple meetings.

**Conclusions:**

Surrogate decisions in Japan are most commonly made by eldest sons and may not frequently consider the perspectives of other surrogates. The finding that patient preferences were rarely known suggests a role for increased advance care planning.

**Supplementary Information:**

The online version contains supplementary material available at 10.1186/s12910-021-00698-0.

## Background

According to one report from the USA, 42.5% of hospitalised older adults need to make decisions about end-of-life treatment. However, only 29.7% of patients have the capacity to make these decisions [1]. In Japan, the national death toll in 2018 was 1.36 million, the highest after World War II, with 70% of these deaths occurring in the 75 + years age group. Since the number of deaths is predicted to increase in the future [2], it is estimated that the number of instances of surrogate decision-making will also increase.

A surrogate decision-maker is a person who makes decisions regarding medical care on behalf of the patient, considering what the patient would want if the patient had the ability to make decisions independently [3]. This is known as the substituted judgement standard. However, the term ‘surrogate decision-maker’ is defined differently across countries. To elaborate, in the United States, the medical law allows the appointing of a ‘medical representative’ for those ‘requiring [a] legal representative’; this legal representative has the right to make medical decisions on behalf of the patient. In contrast, Japan has no such clear law. In reality, the patient’s family members and other people accompanying the patient are currently involved in surrogate decision-making [4]. In this study, those who actually make decisions on behalf of patients are referred to as ‘surrogate decision-makers’, even if they are not legally entitled to make such decisions; moreover, decisions made on behalf of patients are referred to as ‘surrogate decisions’.

There are several guidelines in Japan that should be referred to for surrogate decision-making. Among them, the ‘Guidelines for the Decision-Making Processes in Medical and Long-Term Care for the Elderly’ announced by the Japan Geriatrics Society in 2012, and the ‘Guidelines on the Decision-Making Process for End-Of-Life Care’ revised by the Ministry of Health, Labour and Welfare (MHLW) in 2018 describe the surrogate decision-making processes. Both guidelines posit family members not as surrogate decision-makers but as persons who can presume the patient’s wishes, in case the patient’s preference cannot be confirmed. These guidelines also recommend that decisions should be made together through repeated dialogues between the family members and healthcare team. Regarding the specific steps of this decision-making process, the procedures are described differently depending on whether the preferences and wishes of the patient are understood. The MHLW guidelines indicate that: (1) when the family is able to presume the patient’s wishes, in principle, it is best to respect the presumption; and (2) when the family is unable to presume the patient’s wishes, in principle, it is best to have thorough discussions with them so that the surrogate decision-makers prioritise the patient’s interests. Additionally, the Japan Geriatrics Society recommends consideration of the family’s circumstances in this discussion. There should also be a consensus-building process that involves a multi-disciplinary healthcare team and repeated discussions until a surrogate decision is reached. Since 2015, the MHLW has sponsored a project involving nationwide workshops where its ‘Guidelines on the Decision-Making Process for End-of-Life Medical Care’ are put into practice. These workshops are based on the ‘Education for Implementing End-of-Life Discussion’ programme for medical professionals. The project’s goal is to establish advisory systems at approximately 400 medical institutions throughout Japan to assist with decision-making that respects patient preferences [5]. Some of the issues that have been reported regarding surrogate decision-making to date, both in Japan and internationally, include conflicts between surrogates [6], a high non-congruence rate between surrogate presumptions regarding patient preferences and the patient’s actual wishes [7–9], the inability of some surrogates to follow advanced care planning (ACP) even if it is available [10], and insufficient multi-disciplinary involvement in the decision-making process [11]. However, few large-scale studies have comprehensively examined the entire surrogate decision-making process. In Japan, such a large-scale fact-finding survey is needed to accurately assess the nature and frequency of issues related to surrogate decision-making and explore their trends. By understanding what these issues may be, we might be able to propose ways to use the available guidelines more effectively and develop workshops on surrogate decision-making.

Hence, in preparation for the increased incidences of surrogate decision-making, this study aimed to gather information to assess the current state of surrogate decision-making in Japan, with a focus on how critical decisions are made regarding the use of life-sustaining therapies.

## Methods

This study aimed to assess the current state of surrogate decision-making in Japan.

### Design

A cross-sectional survey was conducted using online questionnaires for surrogate decision-makers in Japan.

### Procedure

A total of 1000 respondents who had experience with surrogate decision-making at Japanese medical institutions were recruited. This survey was outsourced to Cross Marketing Inc. (https://www.cross-m.co.jp/utm_source=google&utm_medium=cpc&utm_campaign=search) and conducted online. Cross Marketing Inc. conducted a survey for monitors owned by a monitor management company (Research Panel, Inc.). The web survey was divided into two parts: a screen that extracted the target participants and a main survey for collecting data. In the screen, individuals registered with the research company were asked whether they had experience in actual surrogate decision-making and to confirm their desire to participate in the survey by answering the questionnaire. Information about the total number of potential participants asked to join the survey and those who met the eligibility criteria was not disclosed to the authors, as per the policy of the company. The collected data were delivered to the authors from Cross Marketing Inc. with ‘Taku file bin’, a file transfer service via web where rigid security management is exercised.

With due regard for the ethical considerations, the first page of the web survey indicated that participation in the survey was voluntary, and participants were presented with the options ‘Agree’ and ‘Disagree’ to indicate their consent to participate in the study. Furthermore, we thought it ethically necessary to consider the mental state of the participants experiencing persistent and significant grief due to their involvement in surrogate decision-making. Therefore, given the sensitivity of targeting surrogate decision-makers, we ensured that a certain amount of time had passed since their participation in the surrogate decision-making process. Thus, only those participants who had acted as surrogate decision-makers at least four months before the time of data collection were invited to participate in our study. We expected the avoidance of investigation immediately after the participants had made their decision as surrogates to have accounted for their grief in the decision-making process and also reduce their mental burden.

This study was approved by the Ethics Committee of Tohoku University Graduate School of Medicine (Approval No. 2019-1-186).

### Participants

The participants judged to be eligible for the study met the following inclusion criteria:

A. The patient for whom the surrogate decision was made was hospitalised between 1 April 2014 and 31 March 2019;
B. The patient had no decision-making capacity for themselves (as judged by their surrogate decision-maker);
C. The patient for whom the surrogate decision was made was 65 years or older;
D. The surrogate decision-maker made clinical decisions regarding life-sustaining treatment including dialysis, artificial respiration, tube feeding, and central venous hyperalimentation, and also participated in meetings with the patient’s physician-in-charge to discuss the treatment options. These meetings took place with individuals such as family members, not the patient themselves.

Participants were excluded from the study if:

A. They did not wish to participate in this study; and
B. They had difficulty communicating in Japanese.

### Questionnaire

The questionnaire for this study was developed by referring to reports of previous academic papers [12], including the result of our preliminary interview survey on the process and judgement grounds of surrogate decision-making in Japan [13]. Questions used in the survey were created by combining the two and examining their validity. Since this study was conducted as a fact-finding survey, the internal validity of the set of the questions was not verified; however, the questionnaire was finalised after a total of five discussions to assure external validity.

An additional file presents details of the questionnaire (see Additional file 1). The questionnaire included items regarding the characteristics of the patients and their surrogate decision-maker (i.e. the respondents); the content of the family meeting between the patient’s physician-in-charge and patient’s surrogate decision-maker, and the latter’s understanding of the former’s explanation; the judgement grounds underlying the surrogate’s decision; and issues relating to the involvement of other parties involved in surrogate decision-making.

#### Characteristics of patients and their surrogate decision-makers (respondents)

Characteristics such as age and gender were investigated with reference to previously published reports on surrogate decision-making [12, 13]. Since it had been reported that patients often expressed their preferences concerning their future treatments verbally as well as in the form of advance directives, and that some patients chose to entrust the decision to others in Japan, we added several questions on this matter [14–16].

#### Family meeting content

Items included were based on the information provided by the doctors to help surrogates reach a decision during the family meeting.

Specifically, during the meeting, it has been recommended that the physician explains the diagnosis, options of treatment, advantages and disadvantages of each option, prognosis, and outcome in case of not undergoing treatment [17]. In addition, a report highlighted the need for doctors to present professional recommendations of options. Since the usefulness of doctors’ sympathetic response to surrogate decision-makers has also been revealed [18], a question on the recognition of surrogate decision-makers by doctors was added in this section.

#### Judgement grounds and others

Many influencing factors have been reported regarding judgement grounds. The items included in the questionnaire mainly comprised those that had been reported in the qualitative survey on judgement grounds in surrogate decision-making based on semi-structured interviews conducted by us prior to this study. A total of 15 items on judgement grounds were included in the questionnaire [19, 20], reflecting the factors related to judgement grounds in surrogate decision-making in Japan and abroad [13, 14]. In addition, the surrogate decision-makers’ behaviour before and after making the decision, the number of family meetings, and the involvement of multiple healthcare professionals were confirmed.

### Scoring

Responses were provided to the questions using five-point and four-point Likert-type scales. The five-point evaluations were applied to the understanding of the family meeting with the healthcare team (*well understood, moderately understood, neither, not well understood, not understood/not explained*) and question from the doctor (*sufficiently, some, neither, not much, almost none*). The four-point evaluations were applied to the involvement of the parties undertaking surrogate decision-making (*directly and heavily, directly, indirectly, littl*e) and judgement grounds (*very emphasised*, *emphasised*, *less emphasised*, *little emphasised/not thinking*).

Dichotomous scoring was used in the analysis (Tables 2 and 4). Responses to the categories of ‘understanding the family meetings’ and ‘question from the doctor’ were provided on a five-point scale; however, in the analysis, a response of ‘neither’ was classified as ‘do not understand’ for the former and ‘not’ for the latter. Responses to the categories of ‘involvement of the parties undertaking surrogate decision-making’ and ‘judgement grounds’ were rated on a four-point scale. However, in the analysis, ‘little’ was classified as ‘not involved’ and the others were classified as ‘involved’ in the former; and ‘very emphasised’ and ‘emphasised’ were classified as ‘emphasised’ and the others were classified as ‘not emphasised’ in the latter.

### Statistical analysis

We describe descriptive statistics and analytical statistics separately. In the descriptive statistics, each variable (e.g. age, gender) is described by a frequency or average value. The distribution is presented as described in the above-mentioned subsection ‘Scoring’. Analysis was performed using bivariate analysis and multivariate analysis. The outcomes were calculated and compared for each group. Among them, for comparison of frequency, chi-square test was performed. Due to the outcome indicated by 0 or 1, the logistic regression model was selected for multivariate analysis. Variables related to attributes were analysed by including items with *P* < 0.20 into the model of multivariate analysis, and the significance level was set to *P* < 0.05. All statistical analyses were performed using STATA v15.0.

## Results

The sample consisted of 1000 people who had had the experience of being a surrogate decision-maker. In order to collect these responses, 80,183 potential participants were screened for having experience as a surrogate decision maker. Among them, 1000 people answered this survey, and the response rate was 100%. Only participants who answered all questions were considered in the final sample. No information was received as to whether there had been participants who did not fully complete the survey.

### Participant characteristics

The participant characteristics are shown in Table 1. Some surrogates were notified in advance of the schedule for a family meeting regarding their decision and did not realise that it meant they had an appointment to discuss the issue before they went to the hospital the next time. In other words, 37.6% of the respondents have no other choice but to make a decision based on a rushed conversation with a medical staff member. No significant differences were found between acute and chronic care hospitals in the number of family meetings required (*P* = 0.25) or the advance setting-up of appointments to discuss the matter (*P* = 0.26). Overall, 48.3% of the patients had communicated their wishes in advance to the surrogate in some form or other.

**Table 1:** The characteristics of surrogate decision-makers and patients

Surrogate decision-makers (n = 1000)	
*Sex*	
Male: 70.5%	
Female: 29.5%	
*Mean age* ± *SD*	
56.29 ± .34 years	
Interquartile range: 50–64 years	
Minimum: 21 years, maximum: 88 years	
*Relationship with the patient*	
Eldest son	48.3%
Eldest daughter	17.7%
Not the eldest child	14.6%
Spouse of offspring (son-in-law or daughter-in-law)	5.8%
Spouse	4.9%
Grandchild	3.3%
Guardian	2.1%
Siblings	2.0%
Others	1.1%
Common-law marriage	.2%
Work during the day	63.7%
Live in the same household with the patient or live close by (can reach patient within 10 min)	46.7%
First experience of surrogate decision-making	89.2%
Patients (n = 1000)	
*Content of the surrogate decision-making*	
Artificial respiration	43.7%
Cardiac massage	14.7%
Dialysis	5.4%
Artificial nutrition (nasogastric tube/intravenous hyperalimentation/gastrostomy)	36.2%
*Treatment location*	
Acute care hospital:	61.1%
Chronic care hospital:	38.9%
Patient’s death (when the surrogate decision-maker responded)	83.9%
*Patient’s ability to communicate*	
Able to communicate	8.6%
Some difficulty	15.3%
Limited to responding to specific requests	17.1%
Not able to communicate at all	59.0%
*Patient's prior preferences*	
In writing	7.6%
Oral	23.0%
Left to the family	15.5%
Left to the doctor	2.2%
Did not leave	48.8%
Do not know	2.9%

### Content of the meetings and its understanding

Table 2 shows whether surrogates could understand the topics covered in the family meetings. Respondents indicated that their family meetings mostly included the information recommended by guidelines in a surrogate decision-making meeting in Japan: the patient’s medical condition, prognosis, treatment options, and the doctor’s recommendations. When asked if the meeting with the doctor had included the aforementioned items, more than 60% of the respondents answered that the doctor had confirmed their understanding, been considerate of their feelings, confirmed the patient’s prior preferences, confirmed how they were able to presume the patient’s preferences, and given their opinion regarding what was best for the patient.

**Table 2:** Content of the surrogate decision-making family meetings

Surrogate decisions (n = 1000)	Understood (%)	Not understood (%)
*Content of the family meetings*		
Medical conditions	96.1	3.9
Treatment options	92.4	7.6
Prognosis without treatment	89.3	10.7
Prognosis with treatment	85.6	14.4
Merits of treatment	83.3	16.7
Concerns about treatment	77.5	22.5
Recommendations of doctors	79.6	20.4

Surrogate decisions (n = 1000)	Included (%)	Not included (%)
Confirmation of understanding	78.3	21.7
Emotional consideration	75.5	24.5
Confirmation of patient’s prior preference	63.7	36.3
How to predict the patient’s preference	62.4	37.6
Doctor’s opinion regarding what is the best for the patient	78	22

When asked to rate how well they had been able to understand the doctor’s explanations regarding the patient’s medical condition, treatment options, prognoses with and without treatment, and the benefits of treatment, more than 80% responded that they had understood them well. However, less than 80% indicated that they had understood the treatment concerns and doctor’s recommendation. In addition, 73.2% of the respondents attended the family meeting with others, 9.9% recorded the family meeting, 44.8% asked the doctor for more time to decide, and 26.7% asked for the explanations in writing.

### Types of participants and their involvement in the decision-making process

Table 3 shows the types of participants potentially involved in the decision process and their level of involvement. The percentages of surrogates and doctors that were directly involved in the decision was, of course, the highest. However, other healthcare providers (i.e. nurses, social workers, and care managers) were not directly involved in most cases.

**Table 3:** Extent of involvement of other parties in the surrogate decision-making process

Surrogate decision-making (n = 1000)	Directly and heavily involved (%)	Directly involved (%)	Indirectly involved (%)	Slightly involved (%)
Surrogate decision-maker	61.1	33.4	5.1	.4
*Family members other than the surrogate decision-maker*				
Resident family	37.1	24.2	12.0	26.7
Non-resident family	18.9	27.2	19.5	34.4
*Healthcare providers*				
Doctor	50.6	30.9	12.1	6.4
Nurse	10.9	26.2	23.9	39.0
Social worker	4.9	10.5	17.4	67.2
Care manager	5.5	9.4	18.8	66.3

### Grounds for surrogate decision-making

Table 4 shows the grounds on which the decision was based and the factors prioritised while making these decisions. For this study, the grounds for judgement were divided into four categories: factors related to the patient’s presumed preference, factors related to the surrogate decision-makers’ preferences, factors related to the patient’s best interests, and other factors.

**Table 4:** Judgement grounds for surrogate decision-making

Surrogate decision-making judgement grounds (n = 1000)	Considered important (%)	Not considered important (%)
*Items related to prediction of the patient’s preference*		
Living will	7.3	92.7
Patient’s presumed preferences	61.8	38.2
Patient’s way of life	86.5	13.5
Patient’s religion	13.4	86.6
*Items related to surrogate decision-maker's preference*		
Surrogate decision-maker’s preference	89.3	10.7
Economic circumstances	39.2	60.8
Long-term care burden	49.9	50.1
*Items related to patient’s best interests*		
Patient's pain	91.1	8.9
Recoverability	88.5	11.5
Possibility of conversation	77.2	22.8
Activities of daily living	76.1	23.9
*Others*		
Consideration for family	42.8	57.2
Consideration for doctor	30.7	69.3
Advice from acquaintance	21.5	78.5
Media information	21.6	78.4

We investigated 15 different factors that could be grounds for basing a surrogate decision. Only 1.8% of the decisions made by the participants in this study were based on a single factor. The rest were based on multiple factors. The most frequently indicated factor was patient suffering (91.1%). Other frequently included factors were those in Categories 2 and 3 (i.e. surrogate preferences and patient’s best interests).

Grouping the responses according to whether the patient had previously left evidence of their wishes in some form, 51.2% of the responses were in the ‘preference in advance’ group and 48.8% were in the ‘no preference in advance’ group. When the responses indicating that the patient’s presumed preferences were used as grounds for making the decision were included in the ‘preference in advance’ group, the difference between this group (71.1%) and the ‘no preference in advance’ group (18.8%) became statistically significant (*P* < 0.001). When the responses indicating that the surrogate’s preferences were used as grounds for making the decision were included in the ‘no preference in advance’ group, the ‘preference in advance’ group (50.0%) became significantly smaller than the ‘no preference in advance’ group (60.7%).

In addition, if the surrogate was the eldest son, 91.7% of the eldest sons included their own preferences in their decision-making, but only 60.2% of the eldest sons included the patient’s presumed preferences.

### The frequency of surrogate decision-making involvement

Table 5 shows the frequency of surrogate decision-making involvement for different stakeholders by number of family meetings (0–1 vs. 2 or more). Of the surrogates, 94.5% were involved, with 61.3% of the cases involving family members living in the same household as the patient and 46.1% involving family members living separately. In terms of healthcare provider involvement, only 37.2% of the cases involved a nurse and only 15.4% involved a social worker despite a doctor being involved in 81.5% of the cases. Further analysis of these data by number of family meetings showed that involvement was significantly lower in the 0–1 family meeting group (n = 261) than in the 2-or-more family meeting group (n = 739) for nurses (*P* = 0.015), social workers (*P* = 0.028), care managers (*P* = 0.011), and family members living separately (*P* = 0.037). This suggested that the opportunity of involvement for more stakeholders may be more limited when there are very few family meetings.

**Table 5:** Frequency of involvement in surrogate decision-making process by stakeholders based on number of family meetings

	0–1 family meeting group (%)	2 or more family meetings group (%)	χ^2^ statistics	*P*-value
*Family members involved in the decision*				
Surrogate decision-maker (n = 945)	143 (95.9)	802 (94.2)	.73	.39
Resident families (n = 613)	82 (55.0)	531 (62.3)	2.90	.09
Non-resident families (n = 461)	57 (38.2)	404 (47.4)	4.34	.037
*Healthcare team members involved in the decision*				
Doctor (n = 815)	118 (79.1)	697 (81.9)	.62	.43
Nurse (n = 372)	42 (28.1)	329 (38.6)	5.96	.015
Social worker (n = 154)	14 (9.3)	140 (16.4)	4.84	.028
Care manager (n = 149)	12 (8.0)	137 (16.0)	6.47	.011

## Discussion

### The surrogate decision-maker’s relationship to the patient

In this study, most surrogate decision-makers were children of patients. The eldest son played that role in 48.3% of the cases; in the United States, this role is usually played by either the spouse or a child [1, 21]. For example, a study of two hospitals in Indiana in the United States found that the surrogates were most commonly daughters (58.9%), sons (25.0%), or spouses (20.6%) [22]. Another Japanese study of patients receiving home medical care found that during ACP, patients named a child, their spouse, or their child’s spouse as their surrogate in 50.9%, 29.6%, and 14.5% of the cases, respectively [14]. Additionally, there may also have been cases in this study in which the surrogate was not the family member the patient named during ACP. It may have been more important to the patient that the surrogate be a child and a male, and thus, be the eldest son. In the following paragraphs, we discuss the reasons behind why these factors may have been important for becoming a patient’s surrogate.

First, children may have played this role rather than the spouse since the spouse of an older adult may also have reduced decision-making abilities. According to the National Institute of Population and Social Security Research [23], the average age of the spouses of older adults is also rising. The age difference between spouses in 1975 was 2.6 years, which had been unchanged from previous years. This may imply that most of the spouses of today’s older adults are possibly not much younger. There is a good reason to assume that patients may have entrusted the role of the surrogate decision-maker to the next generation because their spouses were no longer able to make such decisions or their spouses may have already passed away.

Why is this role most frequently given to the eldest son or another man? The fertility rate in Japan was 4.54 in 1947, 3.65 in 1950, and 1.75 in 1980 [24]. Thus, it is currently likely that an older adult in Japan will have more than one child. Nevertheless, overall, most surrogate decision-makers were either the eldest son or daughter, with 48.3% being the eldest son. In Japan, several norms determine who should be responsible for the care of a parent in their old age or in illness. The determinant factors sometimes used are: being the eldest child, being a son, and living at home. This is referred to as the ‘eldest son/living at home preferential norm’ [25]. There are regional differences in this way of thinking that have changed over time [26]. However, the fact that the eldest son most often played the role of the surrogate in this study suggests the possibility that the selection of a surrogate continues to be based on this patriarchal norm. Taking this into account raises the possibility that the selection process itself could be complicating the surrogate decision-making process, making it more difficult. Based on the substituted judgement standard (what the patient would want if the patient had the decision-making ability) [27], a surrogate should be chosen based on their understanding of the patient’s values and living circumstances. However, if the selection is made based on the above norms without discussion, the surrogate may not necessarily be ‘qualified’ to make those decisions. Such a case could, ethically, be problematic as not only would the surrogate struggle with making these difficult decisions, but the decisions may not reflect the patient’s actual wishes and values. Of course, it is entirely possible that in Japan, the process of selecting a surrogate will change in the future, given that selection is recommended during the ACP process.

### Issues with the decision-making process

The results showed that although the guidelines recommend repeated family meetings, sometimes only one family meeting was held. Moreover, in such cases, significantly fewer decisions involved the participation of healthcare professionals other than the doctor. As previously explained, since 47.4% of surrogate decisions regarding the use of life-sustaining therapies are made within 48 h of hospital admission [22], some decisions must be made within a limited amount of time. However, the reality in Japan regarding the surrogate decision-making process is that sometimes processes considered important in the guidelines are not followed. This is an issue that needs improvement, regardless of any time limitations. Future investigations are needed to devise mechanisms that would enable surrogates and healthcare providers to have repeated discussions, even in situations where time may be limited.

In addition, according to this study’s results, when healthcare professionals other than doctors were involved in the decision-making process, significantly more surrogates indicated that the patient’s presumed wishes were considered as grounds for their decision. The MHLW’s guidelines recommend a process in which adequate discussions are repeated over time between the surrogate decision-maker and the healthcare team to determine what would be in the patient’s best interests when the patient’s wishes cannot be presumed. This healthcare team should be a multi-disciplinary group including long-term care and medical professionals. In addition, it is recommended that the discussion process be repeated over time to respond to changes in the patient’s physical and mental condition and medical assessments. In particular, the importance of these reviews is said to increase towards the end-of-life. In addition, MHLW sponsored workshops utilising the official guidelines entitled ‘Counsellor Workshops on Decision-Making Respecting Patients’ Intentions’ encourage the participation of healthcare professionals from different disciplines in the decision-making process to enable the sharing of values and information as a necessary step in building consensus between the healthcare team and the decision-makers through dialogue [28]. Thus, in the context of surrogate decision-making, multi-disciplinary professional involvement may have encouraged stakeholders to consider how to think and make decisions as if they were the patient.

### Judgement grounds

The grounds on which surrogate decision-makers based their decisions had two features. One feature was that multiple factors were considered. The other was that the factor most frequently considered was the patient’s best interests.

#### Basing decisions on multiple grounds

The survey listed 15 different factors that could be used by surrogate decision-makers as bases for their decisions to see which factor categories were used in each case. Although the study was unable to determine the extent to which each category influenced the surrogate’s decision, it was able to determine the frequency with which each category was considered. In fact, almost all the surrogates based their decisions on multiple grounds rather than just one. A previous study in the United States on a sample of surrogates, 90% of whom were white and female, found that 36% planned to make their decisions using more than one basis [27]. However, the fact that this Japanese study found such a high frequency of surrogates basing their decisions on multiple grounds may well be a characteristic of surrogate decision-making in Japan given the size of the sample.

#### Basing surrogate decisions on factors related to the patient’s best interests

The factor most used by surrogates as the basis for their decision in this study was ‘patient suffering’. This was the leading factor in Category 3 that included factors related to the patient’s best interests and was the most frequently used category for decision-making among participants of this study. Internationally, a study in the United States found that surrogates (also predominantly female and white) focused more on patient well-being than patient preferences as grounds for their decisions [29]. Looking at the frequency with which each basis for decision-making was included from the perspective of the four principles of medical ethics by Beauchamp and Childress (autonomy, non-maleficence, beneficence, and justice), in Japan, surrogates’ considerations may reflect adequate respect for the principles of non-maleficence and beneficence instead of for patient autonomy.

There are two possible explanations for the fact that the patient’s presumed wishes were less frequently included as a basis for decision-making than the patient’s best interests. One could be that it may have been too difficult for the surrogate to guess what the patient’s preferences would have been. The other could be that despite being able to guess the patient’s preferences, the surrogate did not take them into consideration.

First, the low rate of ACP implementation could be a potential reason for difficulty in presuming what the patient’s wishes may be. In this study, only 7.6% of the patients had left a written record of their wishes and 48.8% of the surrogates responded that they did not recognise that the patient had expressed their prior intentions regarding medical care in any form. This is not very different from the percentage of patients who did leave an indication of their wishes (42.2%), according to an MHLW report of 2017 [30]. Hence, it would still be difficult to assert that the number of surrogates or healthcare teams that fully understand the patient’s values regarding the use of life-sustaining therapies has increased.

Another reason for the surrogates finding it difficult to presume what a patient’s wishes might be could be their relative lack of awareness or familiarity with the patient’s daily life compared to the past; as a result, they may have less context to use for reference when they need to make a related decision. The number of older patients living alone has been increasing; furthermore, the number of children living with their older parents has declined dramatically [31]. As a consequence, substituted judgement has become more difficult given how hard it may be for a surrogate to imagine what the patient’s decision would have been at that time in their lives. It is possible that, as a result, surrogates may have abandoned trying to guess what the patient’s wishes would have been, and more frequently based their considerations on the best interests of the patient in accordance with the guidelines.

The next potential explanation is that even though the surrogates were able to presume what the patient’s preferences would have been, they may have ignored these preferences. They may have decided to respect preferences based on Japanese cultural values related to the sanctity of life. According to Asai et al., in Japan, there are deeply ingrained cultural and religious beliefs regarding death and the sanctity of life, and a deeply rooted belief that making the older adults suffer unnecessarily should be avoided [32]. It could be that the high frequency of ‘patient suffering’ (the basis considered most frequently for decision-making in this study) and other factors related to the best interests of the patient were due to cultural and religious values related to consideration for the older adults.

### Research strengths and limitations

This study has two strengths: it is the first large-scale web survey on surrogate decision-making in Japan and it considered responses from the surrogate decision-makers themselves. As for the first strength, the web survey allowed a nationwide grasp of the actual situation. Furthermore, it was possible to comprehensively investigate not only the judgement grounds of surrogate decision-making, but also the processes before and after the decision-making, resulting in a grasp of its characteristics. As for the second strength, it was important that surrogate decision-makers respond to all the survey items, allowing for directly grasping the important recognition of the parties involved in surrogate decision-making. Given the development of discussions based on this recognition, we provided the respondents with important knowledge of future surrogate decision-making.

There are three limitations as well that merit discussion. The first limitation is related to the questionnaire. The questions in this survey included some specialised content. Although explanations to help the respondents understand the questions were added, it is unclear if the answers were accurate. For example, in items asking about the content of the family meeting with the doctor, it is unclear if the respondents were able to accurately classify conversations at the time of the actual family meeting regarding each item (e.g. treatment options, benefits of options, or other options). The second limitation is related to web surveys. First, the response rate is unknown. Additionally, non-Internet users could not answer, and problems related to representation can be noted. It remains possible that patients’ surrogate decision-makers were their older adult spouse and a non-Internet user, further limiting representation. The extent to which the surrogate decision-maker who made the surrogate decision was able to participate in this study is related to the Internet usage rate of the actual surrogate decision-makers, thereby limiting the interpretation of the result. The third limitation is recall bias related to the timing of answering the questionnaire. As for the response period, surrogate decision-makers had the meeting within six months to three years after the surrogate decision-making. Hence, the responses of the surrogate decision-makers may reflect their perception or memory of that time rather than the surrogate decision-making process as it actually occurred at that time.

## Conclusion

In Japan, surrogate decision-makers are commonly the patients’ child, especially the eldest sons. It is questionable to what extent they are aware of the patient's values, have an overall understanding of their lives, and are qualified to be surrogate decision-makers. Moreover, it is also clear that repeated family meetings and the involvement of multi-occupational professionals, both of which are recommended in the guidelines, are not sufficiently carried out in Japan. Furthermore, many surrogate decisions seem to be made after considering multiple judgement grounds in Japan; items related to the patient’s best interests and the preference of the surrogate decision-maker were the judgement grounds considered the most frequently in this study.

Considering the actual situation in Japan, where surrogate decision-making may be limited by time, it may be necessary to devise ways for each person involved in the cross-disciplinary healthcare team to be more actively involved in surrogate decision-making. Further, considering the preferences of surrogate decision-makers, it is necessary to make a surrogate decision after discussion with the healthcare team.

The results of this study showed that in Japan, a multi-disciplinary healthcare team is less likely to be involved in surrogate decision-making when such decisions are based on a one-time family meeting. What we propose is that healthcare professionals from each department be more actively involved and take concrete actions in surrogate decision-making, in which the time to make a decision may be limited. For example, earlier family meetings, efforts to set up repeated family meetings, direct involvement of multi-department health professionals from the first family meetings, and communication with surrogate decision-makers outside of the family meetings may be required. Each of these actions will prevent a situation where doctors and surrogate decision-makers are forced to make surrogate decisions in an isolated manner. Thus, surrogate decisions can then be made through a process of examining judgement grounds with more diverse perspectives and values than is the case currently.

## Supplementary Information

**Additional file 1**. Details of the questionnaire.

## Data Availability

The datasets generated during and/or analysed during the current study are available from the corresponding author on reasonable request.

## Abbreviations

ACP: Advanced care planning
MHLW: Ministry of Health, Labour and Welfare
