# Supplementary material for: Cross-sectional survey of surrogate decision-making in Japanese medical practice
Source: BMC Med Ethics. 2021 Sep 24;22:128. doi: 10.1186/s12910-021-00698-0 (PMC8464150; doi:10.1186/s12910-021-00698-0)
Supplement: Supplementary file 1 — Additional file 1. Details of the questionnaire. [file 12910_2021_698_MOESM1_ESM.docx]

Supplementary

Questions used in the survey were created by combining the two and examining their validity. Since this study was conducted as a fact-finding survey, the internal validity of the set of the questions was not verified; however, the questionnaire was finalized after a total of five discussions in order to assure the external validity.

The details of the questionnaire can be found in the document attached (attached material: questionnaire). The questionnaire included items regarding the characteristics of the patients and their surrogate decision-maker (i.e., our respondents), the content of the interview between the patient’s physician-in-charge and patient’s surrogate decision-maker, and the latter’s understanding of the former’s explanation; the judgment grounds underlying the surrogate decisions; and issues relating to the involvement of other parties involved in the surrogate decision-making.

***Characteristics of patients and their surrogate decision-makers (respondents).*** Characteristics such as age and gender were investigated with reference to previously published reports on surrogate decision-making^12-16^. Since it had been reported that patients often expressed their preferences concerning their future treatments verbally as well as in the form of advance directives and that some patients chose to entrust the decision to others in Japan, we added several questions on this matter.^14-16^

***Interview content.*** The items with reference to the doctor's explanation required for informed consent were established. Specifically, it is recommended that the content of the physician’s explanation in the interview include diagnosis, options of treatment, advantages/disadvantages of each option, prognosis, and outcome in case of not undergoing treatment.^17^ In addition, there was one report that the doctor had better to recommend to present professional recommendations of options. Given a report of usefulness regarding the sympathetic response of doctors to surrogate decision-makers,^18^ the recognition of surrogate decision makers was listed as a question item in this section.

***Judgment grounds and others.*** Many factors have been reported regarding the judgement grounds. Due to cultural differences, the items that had been reported in the qualitative survey on the judgement grounds in surrogate decision-making based on semi-structured interviews^13^, were mainly reflected in the questionnaire. A total of 15 items of the judgment grounds were reflected in the questionnaire,^19,20^ reflecting the factors related to judgment grounds in surrogate decision-making in Japan and abroad.^13,14^ In addition, the behavior before and after the decision of the surrogate decision-maker, the number of interviews, and the involvement of several professionals were confirmed.

***Answer style***

Regarding the items investigating the degree for each answer, we obtained answers in five-point and four-point Likert-type scales. The evaluations were applied to the understanding of the interview (*well understood, moderately understood, neither, not well understood, not* *understood/not explained*) and a question from the doctor (*sufficiently, some, neither, not much, almost none*). The four-point evaluation was applied to the involvement of the parties undertaking surrogate decision-making (*directly and heavily, directly, indirectly, littl*e) and the judgment grounds (*very emphasized*, *emphasized*, *less emphasized*, *little emphasized/not thinking*).

The dichotomy was used in the analysis of the extent of Involvement of Parties in the Surrogate Decision-making Process. The category “involvement of the parties undertaking surrogate decision-making” could be answered on a four-point range: “Little” was classified as “Not involved”, and the others were classified as “Involved.”
